# Health Tract, No. 116

**Published:** 1862-11

**Authors:** 


HEALTH TRACT, No. 116.

### CONSUMPTION.

For just twenty years, there has not been an hour in which I have not had under medical treatment a variety of persons in all the stages of consumptive disease, from the first insidious onset to its last hopeless conditions. With such opportunities, the following observations have been made; nearly all of them but corroborations of eminent medical men in the Old World, as well as the New.

Common Consumption is a gradual and painless destruction of the substance of the lungs, running its course in about two years, when it commences its inroads after twenty-five.

The greatest number who die of Consumption are under twenty-five; the seeds having been planted while in their teens; the effect, mainly, of damp feet, avoidable exposure, inordinate and irregular eating, vicious youthful habits, and bodily irregularities.

Hereditary origin accounts for only about one fourth of the deaths from Consumption, and while it is a disease of all climes and countries, it is comparatively rare in high northern latitudes, and in very elevated localities any where. The city of Mexico, over seven thousand feet above the level of the sea, in latitude nineteen, with a population of about two hundred thousand, gives only three consumptives out of each hundred deaths; while New-York and Boston and London give seven times that number. The seeds of Consumption are called "tubercles," because they look like little tubers or bulbs; each one is a little bunch or "push" on the sides of the air-bladders which make up the lungs; and as none of these are larger than a pea, the tubercles themselves are of necessity much smaller, averaging perhaps the size of a pin-head.

Almost all grown hogs have a kind of hard, tubercular lump in the liver, from one to multitudes, without apparently affecting the health of the animal. So anatomists tell us, that of any hundred dead persons over forty, ninety will show a greater or less number of tubercles in the lungs, although they may have been stricken down in an instant, as in battle, from apparent perfect health. Hence most grown persons have more or less of tubercles; hence, also, a man may have tubercles all his life, and die of old age; therefore it is demonstrable, that tubercles may exist without actual Consumption being present — that is, they are harmless in their quiescent state; as harmless as powder until fire is applied. Tubercles can at any time within three months be excited to a consumptive condition, by one of two causes, and by no other: first, a succession of bad colds; second, by any long debilitated condition of the body, however that debility may have been caused, whether by sickness, grief, over-work of body or brain, or by any depressing influences of long continuance.

A bad cold never can create tubercles, nor can it cause Consumption by any possibility, unless tubercles previously exist. Yet when they do exist, bad colds are the most frequent exciters of Consumption. All the exciting causes under the second head do produce or create tubercles. Hence, the most certain means of avoiding Consumption is, to maintain all the time a high state of general health; and this is almost infallibly done, by living a regular, temperate, active, out-door life. Those who are most out of doors are least liable to Consumption.

The infallible and ever-present signs of Consumption are, a continuation for months of a pulse always beating upward of ninety times in a minute, (seventy being the healthful standard,) thinning of flesh, loss of strength, and an increasing shortness of breath in walking a little fast, up even very slight ascents; there may or may *not* be cough, but nearly always there is a cough on rising or retiring, or both, when more advanced.

An average man, in perfect health of lungs, can blow out at one effort two hundred and fifty cubic inches of air; such a man can not die of common Consumption in a year. This rate gradually diminishes as the decline advances; at one hundred and fifty the case is always and utterly hopeless. The spirometer measures this with infallible and mathematical accuracy. With a pulse of seventy, and a full measurement, the presence of Consumption is an infinite impossibility, however bad the cough or the spitting of blood. The cure of Consumption, when curable—and there are many such cases, is accomplished by an active out-door life, by some sudden and great change of occupation, by the spontaneous appearance of some ulcer or breaking-out on the skin; or by the supervention of some other disease, as asthma, which wards it off and indefinitely postpones it. No medicine or drug, or any thing to be swallowed or inhaled, has ever yet been found which can possibly have any direct, radical, efficient agency in permanently, even arresting the progress of Consumption. Many such have been proposed with great confidence, while all have, one by one, gone out of notice, which could not have been the case had they been efficient. The only means are to secure a vigorous digestion, and to bring back the full breathing of the lungs, both of which are possible

